# Persistent Left Superior Vena Cava Associated with Right Aberrant Subclavian Artery Detected during Totally Implantable Vascular Access Device Insertion

**DOI:** 10.1055/s-0042-1749124

**Published:** 2022-06-14

**Authors:** Etienne El-Helou, Manar Zaiter, Ammar Shall, Youssef Sleiman, Gabriel Liberale, Catalin-Florin Pop

**Affiliations:** 1 Department of Surgical Oncology, Institut Jules Bordet, Université Libre de Bruxelles, Brussels, Belgium; 2 Department of Radiology, Institut Jules Bordet, Université Libre de Bruxelles, Brussels, Belgium

**Keywords:** persistent left superior vena cava, portacath, Hodgkin's lymphoma, case report

## Abstract

**Introduction** Persistent left superior vena cava (PLSVC) is a rare vascular malformation, with several cases reported in the English literature. The diagnosis is made incidentally, during cardiovascular imaging or when a catheter is placed in the left jugular or subclavian vein. They are without associated hemodynamic alterations, except if they have left atrial drainage or an associated dilation of the coronary sinus. If necessary, long-term PSLVC catheterization with right atrial drainage is safe.

**Case Presentation** We report the case of 40-year-old man, admitted for placement of totally implantable vascular access device (TIVAD) on the same day of his first chemotherapy. A disease localized to the right neck made it impossible to puncture on the right. During the puncture of the left internal jugular vein, the diagnosis of PLSVC was made. Postoperative investigations confirmed the diagnosis and showed the presence of the right superior vena cava to which it was connected by the left brachiocephalic vein. They also confirmed the drainage of PLSVC into the coronary sinus. In addition, they demonstrated the presence of an associated right aberrant subclavian artery of direct aortic origin. Chemotherapy was administered safely and the port was removed 9 months after insertion without any problem.

**Conclusion** This is one of the rare cases reported in the English literature of PLSVC diagnosed during TIVAD insertion and the first to report an associated vascular malformation. We publish it to encourage physicians to think about this differential diagnosis and to carefully perform the appropriate investigations before using the port.

Many physicians involved in the treatment of cancer patients, including surgeons, interventional radiologists, and other specialists, routinely use central venous access devices, 1 and they are used to treat a wide range of medical conditions. The administration of drug as chemotherapy, total parenteral nutrition, and poor peripheral venous access are just a few examples. 2

The central venous catheter should be placed over the right ventricle in the superior vena cava (SVC). Due to the rapid flow at this level, thrombogenicity is minimized. 3 When encountering an abnormal guidewire path after a venipuncture, all physicians should be aware of the likely anatomical variations, 4 thus a complete knowledge of the venous anatomy, including the identification of congenital venous abnormalities and treatment- or disease-induced changes in thoracic central venous anatomy, is crucial. 1 4 More than 7% of surgeries result in catheter misplacement, which can lead to life-threatening complications. 3

Persistent left SVC (PLSVC) is a rare vascular variation that is identified by chance during central venous access procedures when the left internal jugular vein (IJV) is used, 5 and it may be associated with other congenital anomalies. 2

Here, we present a case of 40-year-old man, who was diagnosed with PLSVC and right aberrant subclavian artery diagnosed during portacath placement.

## Case Description

A 40-year-old man presented 2 months ago for a lump on the right cervical level, whose ultrasound showed the presence of a right jugulocarotid lymphadenopathy. A biopsy was performed which revealed the presence of a mixed lymphocyte population and the presence of Reed–Sternberg cells, confirming the diagnosis of mixed Hodgkin's lymphoma. Computed tomography and positron emission tomography scan showed disease localized in his right cervical lymph nodes.

Therefore, the patient was scheduled for a totally implantable vascular access device (TIVAD) placement. The patient's preoperative work-up was normal, and he was admitted to the operating room on the same day of his first cycle chemotherapy administration. Intraoperative ultrasound of the neck confirmed the presence of an enlarged right cervical lymph node of 5 cm, with compression of the IJV and consequently inability to puncture the vein. The decision was therefore made for a left catheterization.

Local anesthesia is administered, followed by ultrasound-guided puncture of the left IJV, insertion of the guidewire, and control of its position in the SVC by fluoroscopy, according to our own institutional technique. 6 Intraoperative fluoroscopy showed that the catheter tracks the left mediastinal border. Several attempts to alter the path of the guidewire toward the normal right mediastinal boundary but without success. Considering that the left IJV puncture procedure went smoothly, we concluded that our guidewire follows the path of a PLSVC, and we decided to continue the procedure as usual. After preparation of the subcutaneous area, the dilator was positioned over the guidewire; the catheter and the port were inserted in place, under fluoroscopy control. Rinsing of the port with normal saline, good blood reflux and no flow resistance were noted. Connection of infusion. The procedure went smoothly without difficulty or blood loss, and the patient was transferred to recovery room for further observation and investigations.

**Fig. 1:**
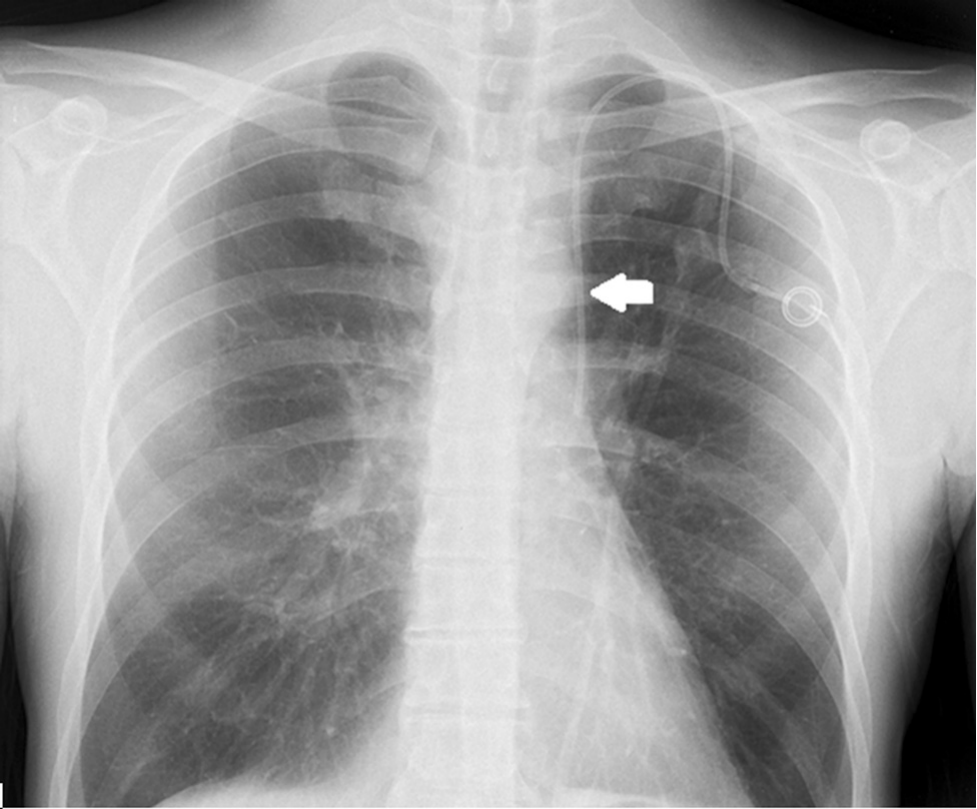
Frontal chest X-ray showing a portacath with left jugular vein approach and demonstrating the unusual course of the catheter in the left hemithorax (arrow), rather than in the right normal anatomical side of the superior vena cava.

The postoperative frontal chest X-ray preformed, demonstrating the unusual path of the catheter in the left hemithorax rather than the normal anatomical right side of the SVC, confirmed the presence of the left SVC ( Fig. 1 ).

**Fig. 2:**
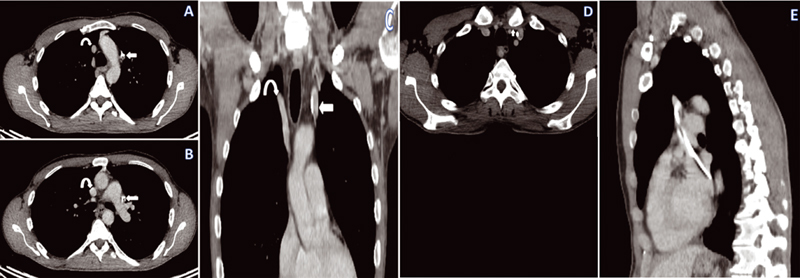
Computed tomography scan. (A, B) Axial view demonstrating the catheter in the persistent left superior vena cava and the right vena cava (curved arrow). (C) Coronal view demonstrating the catheter in the persistent left superior vena cava (arrow) and the right vena cava (curved arrow). (D) Axial view showing the left brachiocephalic vein that communicates the persistent left superior vena cava and the right superior vena cava. (E) Axial view demonstrating the left superior vena cava draining into the coronary sinus.

A computed tomography of the chest was performed to confirm the diagnosis and look for any other associated abnormalities. The following images confirm the presence of the catheter in the PLSVC in addition to the right vena cava in place ( Fig. 2A–C ).

It also highlights the communication between the PLSVC and the right one through the left brachiocephalic vein ( Fig. 2D ), and confirms the drainage of the former into the coronary sinus. Unfortunately, it was difficult to present a clear photo because there is not a large amount of contrast delivered during this chest scan; moreover, the course of the vessel was oblique ( Fig. 2E ).

**Fig. 3:**
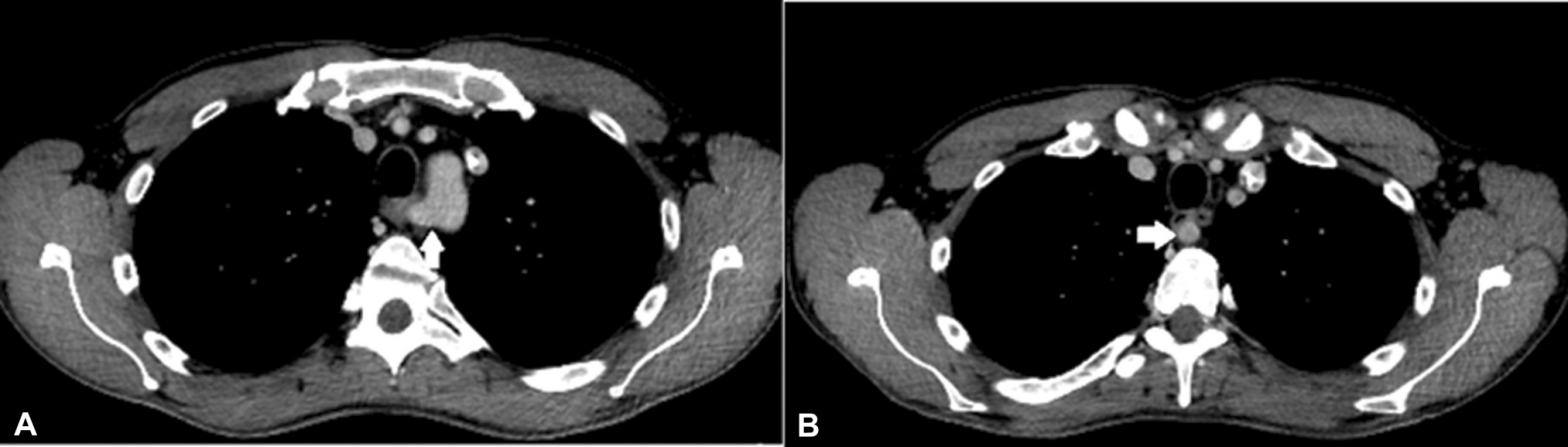
Computed tomography scan: axial oblique views demonstrating the origin of the right aberrant subclavian artery directly from the aortic arch (A) and its relation to the esophagus (B).

Interestingly, another anatomical variant was discovered in this patient; it was an aberrant right subclavian artery that arises directly from the aortic arch, passing posteriorly to the esophagus, compressing it slightly anteriorly and having no correlation with the trachea ( Fig. 3A, B ).

Since the drainage of the PLSVC is confirmed in the coronary sinus and no dysrhythmia developed, the decision was made to start his first dose of chemotherapy, without the necessity of further investigations.

The entire chemotherapy protocol was administered through the portacath without any problem. It was removed 9 months after its insertion.

## Discussion

PLSVC is the most common thoracic venous anomaly, despite its rarity among all vascular malformations. In the general population, the prevalence varies from 0.2 to 3%. In people with congenital heart disease, the prevalence ranges from 1.3 to 11%. 7 Its prevalence does not differ significantly between males and females. 7 The prevalence may be higher if PLSVC was not associated with abnormalities such as hypoplastic thymus and esophageal atresia, 8 which can lead to spontaneous abortions and premature births. 7 There is an increase in associated chromosomal anomalies, the most common association being trisomy 18. 8

John Marshall first reported PLSVC in 1850, 1 and since then, various hypotheses about its development have been suggested. 7 Normally, during the eighth week of embryologic development, a substantial venous anastomosis forms between the left and right precardinal veins, 5 then increased blood flow to the right precardinal vein occurs, allowing it to enlarge. 3 The precardinal veins become the IJVs above this anastomosis. The right precardinal and right common cardinal veins become the SVC below this anastomosis. The left precardinal vein usually regresses, leaving only a small segment as the left superior intercostal vein, while the left common cardinal vein becomes the coronary sinus venous system. 5 The “low left atrial pressure theory” is one of the theories. The left atrium may be smaller than expected in the presence of atrial development anomalies, and it will be unable to fully compress the coronary sinus and left precardinal veins. As a result, the caudal part of the left superior precardinal vein and the left common cardinal vein will not regress, and PLSVC will develop. 7

About 20% of the total return of venous blood from the left arm, left side of the head and neck is handled by PLSVC, and in 80 to 90% of cases, right atrial drainage is found, 7 without associated pathological hemodynamic alterations or clinical symptoms through a dilated coronary sinus. 9 It is symptomatic only if it drains into the left atrium because it bypasses the lungs; it can predispose to systemic dispersion of emboli (right–left shunting), or if conduction problems are caused by an expanded coronary sinus 5 because its expansion may cause compression of the His bundle and atrioventricular node, and in addition, it can cause compression of the left atrium and decreased cardiac output. 7

It is difficult to diagnose on the basis of medical history alone, 3 and physical examination may show distension of the left external jugular vein and/or a heart murmur. 5 A range of cardiac abnormalities affect nearly 40% of people with PLSVC, 10 including associated atrial septal defect, ventricular septal defect, transposition of great vessels, aortic coarctation, and tetralogy of Fallot. 1

The presence and thickness of the two SVCs, the presence of both azygos veins, and the anastomotic ramus were used to classify PLSVC. 11 Evers et al used in simplified classification as follow: type I being the normal anatomy, type II with only a PLSVC and an absent right SVC, type III with the coexistence of the right and left SVCs, which is further subdivided into IIIa where we can find a connection between the two SVCs through the left brachiocephalic vein, while IIIb where the connection between them is absent. 3 The presence of both SVCs, a small anastomotic ramus, and paired azygos veins is the most commonly reported type (20.8%). 11

PLSVC is found incidentally in 75% of cases. 3 It can be identified on chest radiography by local enlargement of the mediastinum superior to the left side of the aortic knob, 12 or during cardiovascular imaging or when a central venous catheter is inserted into the left jugular or subclavian vein. 10 Due to local factors that prevented catheterization or port placement in the right chest, such as breast surgery, radiation therapy, and large metastatic lymph nodes at the root of the right neck, the left side will be chosen. 13

A chest X-ray reveals an atypical catheter path in the left hemithorax. 2 To confirm the diagnosis, computed tomography scan, magnetic resonance imaging, or invasive angiography can be used. 7 Echocardiography is useful to confirm the presence of a dilated coronary sinus and to rule out variations in the typical aberrant venous route. 10 The electrocardiogram can be used to check for cardiac dysrhythmia, 5 manifested by both bradycardia and tachycardia. 4 In about half of patients, PLSVC plays a significant role in the onset and maintenance of atrial fibrillation. 7 Jheengut and Fan used an intracavitary electrocardiogram to identify the PLSVC. A negative P-wave was first observed (in lead II), while a biphasic P-wave pattern appears during catheter insertion. 13 PLSVC draining into the left atrium can be identified by injecting agitated saline into the patient's left arm and observing the timing of left atrial bubbles. 12 Venography is essential to confirm PLSVC drainage into right atrium prior to catheterization when vascular variant anatomy is detected during the procedure. 5

Thermodilution, 3 paradoxical arterial embolism (by air embolus or thromboembolus), 13 coronary sinus thrombosis, venous stenosis, cardiac arrhythmias, cardiac tamponade, and cardiac arrest have all been reported following PLSVC catheterization. 5 Luckily, they are uncommon. 10

Aside from misplacement in a PLSVC, improper placement in minor veins might create an anomalous position of the catheter tip near the left edge of the mediastinum. The left internal thoracic vein, the left superior intercostal vein, the left pericardiacophrenic, 3 the levoatriocardinal vein, and the aberrant left brachiocephalic vein are among them, and they should be considered as differential diagnosis. 7 Misinterpretation can lead to an inaccurate diagnosis of catheter malposition, resulting in unnecessary additional intervention and radiation exposure, as well as delaying the start of essential treatment. 5

Simple ligation, provided that the right SVC is well-developed intracardiac conduit and anastomosis of the PLSVC to the right atrium or the pulmonary artery, can all be used to repair a PLSVC draining into the left atrium. Polytetrafluoroethylene grafts are also used as a venous replacement. 9

When traditional SVC catheterization is not possible, long-term PSLVC catheterization is effective, especially after determining the drainage pattern, 5 and chemotherapy can be administered safely 13 ; ensure that the tip of the catheter does not enter or pierce the coronary sinus, 4 as the presence of the guidewire, dilator, or catheter nearby can cause arrhythmias. 2

After reviewing the English literature, only 17 cases of PLSVC, diagnosed after insertion of a portacath, were reported between 2003 and 2021 ( Table 1 ). The majorities were inserted for chemotherapy administration, and only one was for parenteral nutrition. 10 One of the reported cases had a left atrial drainage and developed a complication post its first usage. 15 None of the reported cases described any associated vascular anomaly.

**Table 1:** Reported cases of PLSVC diagnosed post portacath insertion

Year	Authors	Age	Sex	Comorbidities	Usage	Diagnostic modality	Drain into	Associated vascular anomalies	Used without complications
2003	Laurenzi et al 14	59	M	Lung Ca	Chemo	Chest X-ray	CS	N/A	4 cycles 8 mo
2010	Dinasarapu et al 15	52	F	Breast Ca	Chemo	CT angiogram	LA	N/A	1 cycle (complication post first usage)
2011	Povoski and Khabiri 1	53	F	Breast Ca	Chemo	IO venography	N/A	N/A	7 mo
2012	Iovino et al 10	66	M	Lung Ca	Chemo	Chest X-ray	N/A	N/A	6 cycles
		74	F	Breast Ca	Chemo	Chest X-ray	N/A	N/A	4 cycles
		52	F	Pancreatic Ca	TPN	Chest X-ray	N/A	N/A	2 mo a
		54	F	Ovarian Ca	Chemo	Chest X-ray	N/A	N/A	6 cycles
2016	Zhou et al 5	37	F	NHL	Chemo	IO venography	RA	N/A	8 mo
2017	Evers et al 3	50	–	Esophagus Ca	Chemo	Chest X-ray	CS	N/A	4 cycles NA 4 cycles postop
2018	Van walleghem et al 4	74	M	Lung Ca	Chemo	IO venography	CS	N/A	N/A
2021	Jheengut and Fan 13	46	F	Breast Ca	Chemo	ECG	N/A	N/A	6 cycles 4 mo
		41	F	Breast Ca	Chemo	ECG	N/A	N/A	4 cycles 3 mo
		33	F	Breast Ca	Chemo	ECG	N/A	N/A	20 cycles 25 mo a
		59	M	Lung Ca	Chemo	ECG	N/A	N/A	20 cycles 7 mo a
		57	F	Breast Ca	Chemo	ECG	N/A	N/A	6 cycles 6 mo
		41	M	Lung Ca	Chemo	ECG	N/A	N/A	6 cycles 16 mo b
		51	M	Lung Ca	Chemo	ECG	N/A	N/A	6 cycles 13 mo b

Abbreviations: Ca, cancer; Chemo, chemotherapy; CS, coronary sinus; CT, computed tomography; ECG, electrocardiographic; F, female; IO, intraoperative; LA, left atrium; M, male; N/A, not available; NA, neoadjuvant; NHL, non-Hodgkin's lymphoma; Postop, postoperation; RA, right atrium; TPN, total parenteral nutrition.

a Patient died before stop usage.

b Patient is still using his port at the time of publication.

## Conclusion

PLSVC is a rare congenital vascular malformation, detected incidentally in the majority of cases and can lead to morbidity and mortality during its puncture and use. We urge all physicians to keep this differential diagnosis in mind and carefully revise any available preoperative imaging that may help detecting any vascular anomalies such as PLSVC.

We reported here the first case of association of vascular anomalies in patient where we discovered a PLSVC during the placement of TIVAD.
